# Cholangitis and Interruptions of Neoadjuvant Chemotherapy Associate with Reduced Overall and Progression-Free Survival in Pancreatic Cancer

**DOI:** 10.1245/s10434-023-14793-6

**Published:** 2023-12-28

**Authors:** Sini Vehviläinen, Antti Kuuliala, Marianne Udd, Anna Nurmi, Katriina Peltola, Caj Haglund, Leena Kylänpää, Hanna Seppänen

**Affiliations:** 1 Department of Gastrointestinal Surgery, Meilahti Hospital, Helsinki University Hospital, Helsinki, Finland; 2 Translational Cancer Medicine Research Programme, University of Helsinki, Helsinki, Finland; 3 Bacteriology and Immunology, University of Helsinki and Helsinki University Hospital, Helsinki, Finland; 4 Department of Oncology, Helsinki University Hospital, Helsinki, Finland

**Keywords:** Cholangitis, Pancreatic cancer, Survival, Neoadjuvant treatment, Biliary decompression

## Abstract

**Background:**

Interrupting chemotherapy may explain the reduced overall survival (OS) in patients with pancreatic cancer (PC) with cholangitis. Endoscopic biliary decompression (BD) with metallic stents results in fewer chemotherapy interruptions and a lower cholangitis rate compared with plastic stents. We aimed to determine the impact of cholangitis, neoadjuvant treatment (NAT) interruptions and biliary stent choice on PC patients’ survival.

**Methods:**

We conducted a retrospective analysis of 162 patients with cancer of the head of the pancreas undergoing pancreatoduodenectomy after NAT and BD documenting progression-free survival (PFS) and OS. Data on BD, cholangitis, stent type, surgical radicality, and chemotherapy were collected. Survival was estimated based on the Kaplan–Meier method by using the log-rank test and multivariate Cox regression analysis.

**Results:**

Median OS and PFS for patients with cholangitis (*n* = 33, 20%) were 26 and 8 months (95% confidence interval [CI] 20–32 and 5–10 months), respectively, compared with 36 and 17 months (95% CI 31–41 and 12–21 months; *p* < 0.001 for OS; *p* = 0.002 for PFS) for patients without cholangitis. Among patients without NAT interruptions median OS and PFS were 35 and 17 months (95% CI 31–40 and 12–21 months), falling to 26 and 7 months (95% CI 18–30 and 5–10 months) among those who experienced an NAT interruption caused by biliary stent failure (n = 26, 16%) (*p* = 0.039 for OS; *p* < 0.001 for PFS). We found no difference in OS or PFS between stent types.

**Conclusions:**

Cholangitis and NAT interruptions reduce OS and PFS among PC patients.

**Supplementary Information:**

The online version contains supplementary material available at 10.1245/s10434-023-14793-6.

Patients with pancreatic cancer (PC) experience a poor overall survival (OS). Globally, the 5-year survival rate ranges from 2 to 11.5%.^1–5^ Surgical resection together with oncological treatment remains the only option for a cure. The 5-year survival rate among successfully operated on patients ranges from 20 to 27%.^1,4–8^ Only 8–20% of all PC patients present with initially resectable or borderline resectable (BRPC) tumours eligible for radical surgery,^4,8–10^ whereas the majority of patients present with locally advanced (LAPC) or metastatic unresectable disease. Among nonsurgical patients, the median survival from diagnosis ranges from 2 to 6 months in metastatic disease and 6–11 months for locally advanced PC.^1^

Expert consensus statements and meta-analyses recommend a systemic chemotherapy approach preceding surgery for BRPC and LAPC.^11–13^ A recent systematic review reports a resectability rate of 60% for BRPC after NAT.^14^ In the same review, the resectability rate for LAPC after NAT was 22%.^14^ Recent studies suggest improved progression-free survival (PFS) rates after NAT for patients with resectable pancreatic tumours.^15–19^ There is a lack of consensus on the superiority of one NAT regimen against another. Weak evidence suggesting improvement in survival and resectability rates after NAT with combination of leucovorin, fluorouracil, irinotecan, and oxaliplatin (Folfirinox) has been reported.^20^ Concerning adjuvant chemotherapy in resectable PC, Folfirinox regimens have indicated improvement in OS and PFS rates with the expense of higher incidence of toxic events.^21^

Cholangitis might represent an independent risk factor for reduced OS in PC patients.^22^ Furthermore, pancreatobiliary cancer patients with cholangitis have higher 28-day mortality compared with noncancer cholangitis patients.^23^ Perhaps, patients who present with cholangitis have a more aggressive type of PC.^22^ In our experience, episodes of cholangitis cause interruptions to NAT, possibly contributing to the reduced OS and PFS. The impact of interrupted NAT on survival among surgical PC patients remains undocumented.

Cholangitis is managed with antibiotics and endoscopic biliary decompression. Studies comparing self-expandable metallic stents (SEMSes) and plastic stents (PSes) for biliary decompression demonstrate that PC patients with SEMS have a lower rate of cholangitis, fewer stent exchanges, and fewer chemotherapy interruptions.^24–28^ Guidelines for endoscopic drainage recommend biliary decompression withs SEMS in PC cases with a confirmed histology.^29^ The question regarding the effect of the biliary stent type on OS and PFS is unanswered.

In this study, we hypothesised that cholangitis and NAT interruptions would diminish OS and PFS in BRPC and LAPC patients undergoing pancreatoduodenectomy after NAT. We aimed to determine the association between cholangitis, NAT interruption, and a reduced survival. Secondarily, we aimed to identify the differences in survival rates between patients with different stent types used for biliary decompression, administered chemotherapy regimens, and surgical radicality.

## Methods

This retrospective, single-centre study was conducted at Helsinki University Hospital (HUH). We searched the HUH database to identify patients who underwent pancreatoduodenectomy in HUH due to an adenocarcinoma in the head of the pancreas between 2000 and 2022. Only patients undergoing NAT (in HUH or other hospital districts) and endoscopic biliary decompression before surgery were included. The HUH administration granted their approval to search the local patient database. All data were collected in a retrospective manner. Therefore, consent from the enrolled patients or approval from the hospital ethics board was not required.

Dates for diagnosis, endoscopic retrograde cholangiopancreatography (ERCP), pancreatoduodenectomy, preoperative cholangitis, and other clinically significant infections affecting chemotherapy and PC recurrence were collected from medical charts. The Finnish Population Information System provided verification of the time of death or whether the patient was still alive. The date of diagnosis was defined as the date when the pancreatic tumour was first detected via a computed tomography (CT) scan. The time of recurrence was defined as the date when a local recurrent tumour, peritoneal carcinosis, or distant metastasis was detected via a CT scan.

We collected additional data on general patient characteristics (age, gender, Charlson Comorbidity Index [CCI], and comorbidities), PC characteristics (preoperative carcinoembryonic antigen [CEA] and carbohydrate antigen 19-9 [CA19-9] values and TNM-classification^30^ from pathology reports), and treatment characteristics (preoperative treatment, major interruptions to chemotherapy, adjuvant chemotherapy regimens, surgical radicality, biliary stent type [PS or SEMS], and endoscopy- and surgery-related complications). A major interruption to chemotherapy was defined as a discontinuance of chemotherapy for more than 28 days or a premature termination of chemotherapy.

Cholangitis was defined by using the presentation of clinical symptoms. In all cases, clinical symptoms included elevated plasma bilirubin levels (>40 μmol/l). Additionally, clinical symptoms included an elevated body temperature (>38.0 °C) and/or an elevated plasma C-reactive protein (CRP) level (>50 mg/l) combined with a thickening of the biliary duct walls visible on a CT scan or visually infected biliary fluid observed during endoscopy. Biliary obstruction without cholangitis was defined as elevated plasma bilirubin levels (>40 μmol/l) without symptoms of infection.

For biliary decompression, plastic stents consisted of Tannenbaum stents (5–7-cm long) with a diameter of 10 Fr. The majority of SEMS used were 6–8-cm-long WallFlex stents (Boston Scientific) or Hanaro stents (Olympus Medical) with a diameter expanding up to 10 mm. Only seven of the SEMS used were covered.

Surgical radicality was evaluated from the resection margin reported in the pathology report. R0 resection was defined as a ≥1-mm, disease-free margin from the tumour in all directions. As key outcomes, we calculated the OS and PFS of patients undergoing pancreatoduodenectomy for PC. We analysed several patient- and surgery-related factors thought to impact these outcomes. Complications related to pancreatic surgery requiring endoscopic, surgical, or radiological intervention (Clavien-Dindo III-IV) and ERCP (Cotton consensus criteria) were documented.^31,32^

### Statistical Analysis

Data are reported as the number of cases with the percentage or as a median value with the range or 95% confidence interval (CI). To determine the dependency of two categorical variables, we tested for statistical significance by using the Fisher’s exact test. For continuous variables, we used the Mann-Whitney *U* test. Survival was estimated by using the Kaplan–Meier method with log-rank test and with univariate and multivariate Cox regression. OS was calculated from the date of diagnosis, and PFS from the date of surgery. We considered *p* ≤ 0.05 statistically significant, and no adjustment was made for multiple testing. All statistical analyses were calculated by using SPSS version 27 (IBM, Armonk, NY).

### Results

In total, 162 pancreatoduodenectomies were performed following NAT on Finnish patients. At the time of diagnosis, 144 (89%) patients presented with BRPC and 18 (11%) with LAPC. Vascular resection with reconstruction was required in 48 (30%) patients. The median OS among all patients was 33 (95% CI 29–37) months, whereas PFS was 13 (95% CI 10–17) months. There was no difference in OS (log-rank, *p* = 0.074) between patients with BRPC (median 35 months, 95% CI 31–39 months) and LAPC (median 27 months, 95% CI 15–37 months). However, there was a statistically significant difference in PFS (log-rank, *p* = 0.045) between BRPC (median 14 months, 95% CI 9–19 months) and LAPC (median 7 months, 95% CI 5–10 months). At the time of data analysis on 30 June 2023, 49 (30%) patients were still alive, 29 (59%) of whom were cancer-free at the most recent follow-up. Due to follow-up in another hospital district, recurrence data for 39 (24%) patients were unavailable. The median time from diagnosis to surgery was 5.8 (range 2.5–16.4) months. Table 1 summarizes the patient characteristics. Age, gender, CCI, a history of diabetes or another cancer, time from diagnosis to surgery, and ASA classification all had no significant impact on OS or PFS.

**Table 1:** Patient characteristics

	All (*n* = 162)	Cholangitis (*n* = 33)	No cholangitis (*n* = 129)	NAT interruptions due to stent (*n* = 26)	Other NAT interruptions (*n* = 8)	No NAT interruptions (*n* = 128)
*Mean (SD)*						
Age (years)	66.0 (7.8)	69.8 (7.1)	65.3 (7.8)	67.3 (6.6)	60.7 (9.1)	66.1 (7.9)
CCI	4.6 (1.2)	4.9 (1.3)	4.6 (1.2)	4.8 (1.3)	3.8 (0.9)	4.6 (2.1)
*Median (IQR)*						
Ca19-9 (U/mL)	59 (244)	241 (1263)	35 (123)	744 (1329)	30 (122)	46 (156)
CEA	2.4 (2.1)	2.3 (2.0)	2.4 (2.1)	2.5 (2.7)	2.0 (1.5)	2.7 (4.0)
*n (%)*						
Male	83 (51)	13 (39)	70 (54)	12 (46)	1 (13)	70 (55)
Female	79 (49)	20 (61)	59 (46)	14 (54)	7 (87)	58 (45)
Diabetes	36 (22)	4 (12)	32 (25)	3 (12)	2 (25)	31 (24)
Prior malignancy	12 (7.4)	1 (3.0)	11 (8.5)	1 (3.8)	0	11 (8.6)
*ASA*						
I	6 (3.7)	1 (3.0)	5 (3.9)	0	0	6 (4.7)
II	83 (51)	16 (48)	67 (52)	14 (54)	7 (87)	62 (48)
III	69 (43)	13 (39)	56 (43)	10 (38)	1 (13)	58 (45)
IV	4 (2.5)	3 (9.0)	1 (0.7)	2 (7.7)	0	2 (1.6)

*NAT* neoadjuvant chemotherapy; *CCI* Charlson Comorbidity Index; *Ca19–9* serum carbohydrate antigen 19–9; *CEA* serum carcinoembryonic antigen; *IQR* interquartile range; *ASA* American Society of Anesthesiologists’ physical status classification

### Cholangitis

**Fig. 1:**
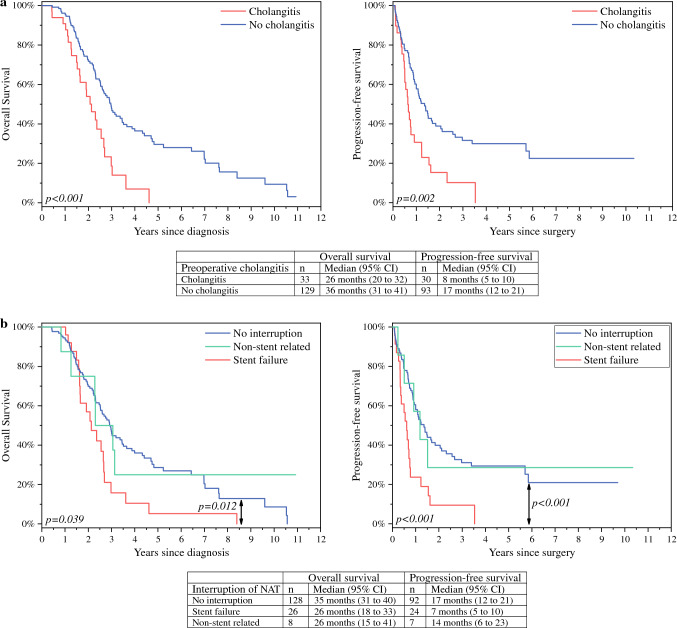
Median overall and progression-free survival times in 162 patients undergoing a pancreatoduodenectomy with or without preoperative cholangitis (**a**) and neoadjuvant chemotherapy interruptions (**b**). Recurrence data were available for 123 patients. Kaplan-Meier analysis for overall (left) and progression-free survival (right). Log-rank test *p*-values are shown at the bottom-left corners, and statistically significant post hoc* p*-values between groups are indicated by arrows. NAT, neoadjuvant chemotherapy

Among all patients, 33 (20%) experienced at least one episode of cholangitis requiring an endoscopic intervention, antibiotic treatment, and hospitalisation. We detected a statistically significant difference in OS (*p* < 0.001) and PFS (*p* = 0.002) between patients with at least one episode of cholangitis and those patients without (Fig. 1a). Cholangitis was associated with a delayed surgery, where the median time from diagnosis to surgery among patients with one or more episodes of cholangitis was 7 months, falling to 5.7 months for patients without cholangitis (*p* = 0.048).

### Interruption of Neoadjuvant Chemotherapy

NAT was administered without interruption to 128 (79%) patients with a median OS of 35 (95% CI 31–40) months and a median PFS of 17 (95% CI 12–21) months. We divided NAT interruptions into two subcategories: interruption due to biliary stent failure (cholangitis or stent occlusion without cholangitis) and nonstent-related reasons (including cholecystitis, neutropenic sepsis, and a severe allergic reaction to irinotecan). Patients with a NAT interruption due to stent failure had a lower OS and PFS compared with patients with no NAT interruptions (for OS *p* = 0.012; for PFS *p* < 0.001; Fig. 1b). However, OS and PFS did not differ significantly between patients experiencing no NAT interruptions and patients with a nonstent-related interruption.

### Biliary Stent Type

The median time from diagnosis to the first ERCP and biliary drainage was 5 (range 0–148) days. A PS was placed as the primary stent in 144 (89%) patients. In 18 (11%) cases the first stent was SEMS. Indications for a primary stent consisted of jaundice without cholangitis in 153 (95%) cases and cholangitis in nine cases (5%). None of the patients with primary SEMS needed a repeat ERCP before surgery. Among patients with a primary PS, 55 (34%) underwent a stent exchange (11 routine, 24 for cholangitis, and 20 for stent failure without cholangitis). Stent failure rates for the first biliary stent were 0% for SEMS and 31% (*n* = 44) for PS (*p* = 0.020). Among 55 exchanged PSes, 34 (62%) were replaced with a SEMS and 21 (38%) with a PS. Furthermore, among 21 patients with a second PS, five (24%) patients underwent an additional stent exchange (four routine, one stent migration). Among 34 patients with a SEMS as the second stent, two (5.8%) patients had an additional repeated ERCP due to a stent occlusion. In total, the 162 PC patients underwent 224 ERCPs with a total stent failure rate of 21% (*n* = 47). Interestingly, all cases of stent dysfunction or cholangitis do not cause clinically significant NAT delays. The relationship between stent dysfunction, cholangitis and clinically significant NAT interruptions is presented in Table 2.

**Table 2:** Stent failure and NAT interruptions in 162 PC patients with a plastic or self-expandable metallic stent

	NAT terminated or interrupted (*n*)%	No major interruption, or NAT was completed before stent failure (*n*)%
Stent failure and cholangitis: 24 (15%) patients*	19 (79%)	5 (21%)
Stent failure without cholangitis: 20 (12%) patients**	7 (35%)	16 (65%)

^*^One patient later experienced an additional episode of cholangitis

^**^Three patients experienced an additional episode of stent dysfunction without cholangitis

*NAT* neoadjuvant chemotherapy

**Fig. 2:**
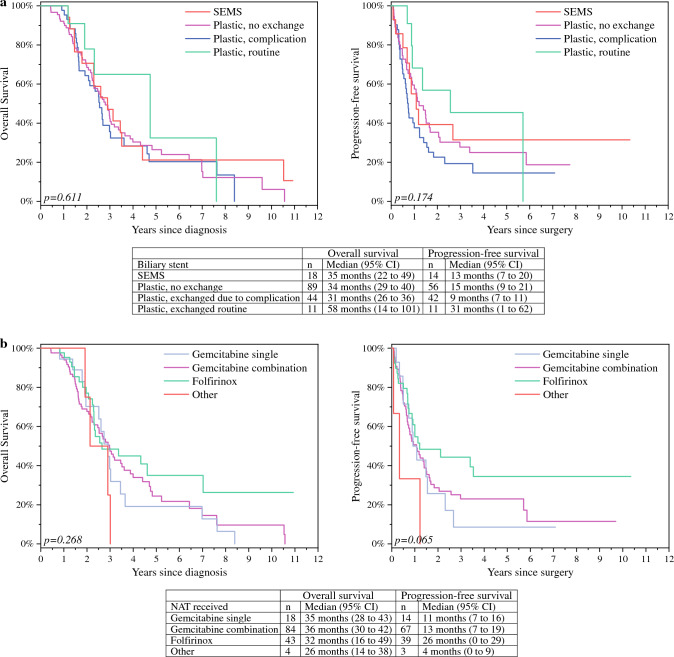
Biliary stents used for biliary decompression (**a**) and neoadjuvant chemotherapy received (**b**) in patients undergoing a pancreatoduodenectomy. Median overall and progression-free survival times are shown. Data on neoadjuvant regimens were available for 149 patients, 123 of whom had data on recurrence. Kaplan-Meier analysis for overall (left) and progression-free survival (right). Log-rank test pvalues are shown at the bottom-left corners, and statistically significant post hoc *p*-values between groups are indicated by arrows. Abbreviations: NAT, neoadjuvant chemotherapy; SEMS, self-expandable metallic stent

We analysed the difference in median OS and PFS between patients with only one biliary stent placed before surgery compared with those who had stent exchanges routinely or due to stent complications. Stent type (PS or SEMS) was taken into account. We detected no difference in OS or PFS between stent type groups (for OS *p* = 0.610, for PFS *p* = 0.174; Fig. 2a).

### Neoadjuvant Chemotherapy

Given that preoperative treatment was provided by various hospitals, the NAT regimens for 13 (8.0%) patients remained unknown. We analysed the following subgroups separately: (1) gemcitabine alone; (2) gemcitabine in combination; (3) Folfirinox; and (4) other regimens. Figure 2b presents the median OS and PFS for the different NAT regimen groups. We detected no statistically significant differences in OS or PFS between patients based on different neoadjuvant regimens (for OS *p* = 0.268; for PFS *p* = 0.065). For detailed data on the neoadjuvant treatment administered, see Supplemental Table 1.

### Adjuvant Chemotherapy

A detailed list of the adjuvant chemotherapy administered appears in Supplemental Table 1. Data on adjuvant chemotherapy were missing for 20 (12%) patients given that oncological follow-up care was provided in another hospital district, whereas 34 (21%) patients did not receive adjuvant chemotherapy. We analysed the adjuvant chemotherapy subgroups (gemcitabine alone, *n* = 40 (25%); gemcitabine in combination *n* = 52 (32%); Folfirinox *n* = 15 (9.2%); and other regimens, *n* = 1) separately. We detected no statistically significant differences in OS (for gemcitabine alone: 35 [95% CI 32–38] months; gemcitabine in combination: 30 [95% CI 22–38] months; Folfirinox: 32 [95% CI 23–41] months; other: 128 months [95% CI NA]; no adjuvant therapy: 32 [95% CI 23–37] months; *p* = 0.256) or PFS (for gemcitabine alone: 12 [95% CI 8–16] months; gemcitabine in combination: 11 [95% CI 5–17] months; Folfirinox: 9 months [95% CI NA]; no adjuvant therapy: 18 [95% CI 16–21] months; *p* = 0.266) between adjuvant chemotherapy groups.

Of the 108 patients who were known to receive adjuvant chemotherapy, the exact date for initiation of adjuvant chemotherapy was available for 78 (72%). Median time from surgery to initiation of adjuvant chemotherapy was 54 (range 25–174) days. For six patients (7.7%), adjuvant chemotherapy was initiated more than 90 days after surgery (median 114 days, range 93–142). There was no statistically significant improvement in OS (*p* = 0.959) or PFS (*p* = 0.811) in patients with adjuvant chemotherapy initiation within 90 days of surgery compared to other patients.

### Tumour Stage

**Fig. 3:**
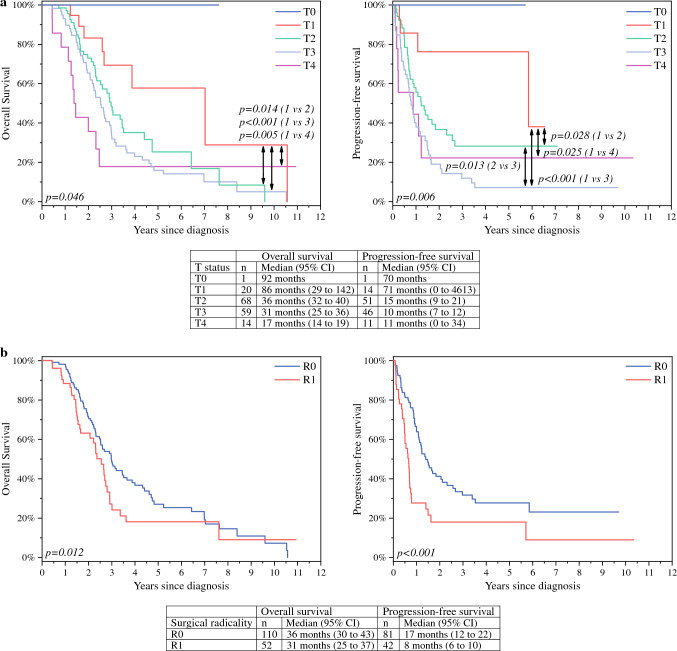
Tumour staging (**a**) and surgical radicality (**b**) in 162 patients undergoing a pancreatoduodenectomy. Median overall and progression-free survival times are presented. Recurrence data were available for 123 patients. Kaplan-Meier analysis for overall (left) and progression-free survival (right). Log rank test *p*-values are shown in bottom-left corners, and statistically significant post hoc *p*-values between groups are indicated by arrows.

T status was verified from postoperative pathology reports. We established a statistically significant association between a higher T stage and both lower OS and PFS (for OS *p* = 0.046; for PFS *p* = 0.006; Fig. 3a). The number of patients in different T stage groups and their median OS and PFS appear in Fig. 3a.

The N status of the nearby lymph nodes was checked via pathology reports from surgical specimens. A significant trend indicated a lower OS as the N status increased (for N0: 42 [95% CI 34–50) months; N1: 32 [95% CI 28–36] months; N2: 24 [95% CI 14–35] months; *p* = 0.008). For PFS, the trend was not statistically significant (for N0: 18 [95% CI 12–25] months; N1: 12 [95% CI 8–16] months; N2: 8 [95% CI 5–11] months; *p* = 0.095).

### Surgical Radicality

The R0 resection rate was 67%. We detected no significant difference in OS between patients with R0 or R1 surgical radicality (*p* = 0.10). Yet, a small but significant advantage was noted in PFS favouring R0 resections (*p* = 0.001; Fig. 3b).

### Univariate and Multivariate Cox Regression for OS and PFS

The univariate Cox regression analysis detected a statistically significant reduction in OS among patients with preoperative cholangitis, higher T and N tumour stages and among those who experienced an interruption in NAT due to stent failure, but not among those with nonstent-related interruptions. Comparing the different chemotherapy regimens, biliary stent types, BRPC versus LAPC or surgical radicality revealed no significant differences in the hazard ratios (HRs) in the univariate analysis. We then conducted a multivariate Cox regression analysis entering the statistically significant covariates resulting from the previous univariate analysis. The multivariate analysis showed statistically significant reduction in OS in patients with preoperative cholangitis and higher T and N tumour stages (Table 3). Respectively, the Cox univariate analysis indicated a statistically significant reduction in PFS among patients with preoperative cholangitis, higher T tumour stage, NAT interruptions, and R1 resections. Patients with higher T stage and R1 resections exhibited a lower PFS in the multivariate Cox regression analysis. However, the multivariate analysis found no significant difference in PFS comparing patients with or without cholangitis or NAT interruptions (Table 3).

**Table 3:** Univariate (only statistically significant variables shown) and multivariate Cox regression for overall and progression-free survival

	Univariate			Multivariate		
	HR	95% CI	*p*	HR	95% CI	*p*
*Overall survival*						
Preoperative cholangitis	2.28	1.44–3.62	< 0.001	2.06	1.08–3.91	0.028
Interruptions of NAT						
None	(1.00)			(1.00)		
Due to stent	1.83	1.13–2.97	0.014	0.93	0.47–1.87	0.84
Due to other	0.88	0.38–2.06	0.77	0.67	0.26–1.71	0.40
T-status*						
1	(1.00)			(1.00)		
2	1.99	0.93–4.27	0.078	1.94	0.89–4.23	0.098
3	2.68	1.27–5.65	0.010	2.43	1.13–5.20	0.023
4	3.24	1.29–8.14	0.012	3.25	1.19–8.88	0.021
N-status						
0	(1.00)					
1	1.48	0.99–2.23	0.058	1.51	1.00–2.30	0.053
2	2.60	1.38–4.90	0.003	2.10	1.07–4.13	0.032
*Progression-free survival*						
Preoperative cholangitis	2.10	1.30–3.37	0.002	0.86	0.40–1.86	0.71
Interruptions of NAT						
None	(1.00)			(1.00)		
Due to stent	2.56	1.54–4.27	< 0.001	1.23	0.46–3.27	0.68
Due to other	1.03	0.41–2.57	0.95	1.87	0.51–6.86	0.347
T-status*						
1	(1.00)			(1.00)		
2	2.92	1.03–8.25	0.044	2.94	1.03–8.39	0.043
3	5.04	1.80–14.10	0.002	4.57	1.63–12.85	0.004
4	4.31	1.26–14.73	0.020	3.60	0.97–13.35	0.055
R0 resection	2.14	1.37–3.33	< 0.001	1.93	1.17–3.19	0.011

^*^One patient with T0 omitted from analysis

*HR* hazard ratio; *NAT* neoadjuvant chemotherapy; *CI* confidence interval; *HR* hazard ratio

### Complications Related to ERCP and Pancreatoduodenectomy

In total, 226 ERCPs were performed on 162 patients. We observed no severe complications related to ERCP. Post-ERCP pancreatitis rate was 3.0% (*n* = 7). In total, ten (6.2%) patients needed a repeat laparotomy within 90 days of surgery. Complications related to ERCP and pancreatoduodenectomy are presented in Supplemental Table 2.

## Discussion

The median OS of 33 months and PFS of 14 months found in this study agree with previously reported studies on surgical NAT patients with PC.^1,19,33^ As previously suggested, we identified an association between preoperative cholangitis and a lower survival after pancreatoduodenectomy.^22^ A previous study suggested that with proper biliary decompression cholangitis does not impact PC patients’ ability to complete NAT.^22^ In our study, NAT interruptions were indeed primarily caused by cholangitis or a biliary stent occlusion, and we found a statistically significant relationship between NAT interruption and lower survival. Interestingly, the reduction in OS and PFS in this study only appeared to accompany NAT interruptions due to biliary stent complications. Interruptions due to nonstent-related issues did not reduce OS or PFS. Thus, we argue that cholangitis impairs patients’ chances of undergoing uninterrupted NAT and that interrupted NAT associates with lower overall survival.

With rates reported up to 30% biliary stent failure is an issue among PC patients during NAT.^34^ For jaundiced patients eligible for upfront surgery an imminent surgery without biliary decompression should be considered.^35^ Proceeding to upfront surgery spares the patient from stent-related complications. For patients with cholangitis or NAT for LAPC/BRPC biliary decompression is essential despite the risk of stent failure. The total stent failure rate of 21% in this study falls in the range of prior demonstrations.^34^ Previous studies indicate that PC patients have a lower rate of preoperative cholangitis and NAT interruptions when SEMSes are used for biliary decompression.^24–28^ Our results mirror these findings and therefore support guidelines recommending SEMS for biliary decompression of malignant biliary obstruction.^29^ We found no association between SEMS and an improved survival. One reason could be that the number of patients obtaining a primary SEMS in this study was low.

The PREOPANC trial reports an issue with persistent jaundice after biliary drainage causing unspecified delay to NAT in ten patients.^36^ In this study, no clinically significant delays in NAT due to persistent jaundice after biliary decompression were observed. To prevent NAT delays, we perform repeat ERCP within 14 days if the patient’s serum bilirubin level is not decreasing by 50% and imaging indicates ongoing biliary obstruction. Plastic stents are replaced with covered or uncovered SEMSes. It is unclear which stent type was used among the ten patients that PREOPANC trial reported and in which timeframe possible repeat ERCP was performed. Based on previous knowledge we suggest that in resectable PC with persistent jaundice, immediate surgery without further biliary decompression attempts should be considered.^35^

For adjuvant chemotherapy, patients receiving Folfirinox and gemcitabine-capecitabine combinations may experience better long-term survival after pancreatic surgery compared with patients receiving gemcitabine monotherapy.^9,21,37^ Evolving data suggest that Folfirinox should be the standard therapy in PC patients fit for adjuvant treatment after a pancreatic resection.^38^ In our study, we detected no difference in survival rates between various NAT or adjuvant chemotherapy regimen groups. This discrepancy with previous studies on adjuvant chemotherapy may be due to the variability in the selected chemotherapy resulting from our long inclusion period and from chemotherapy being administered in various hospitals. A significant portion of our patients were treated in the era preceding the introduction of Folfirinox as NAT or adjuvant chemotherapy for PC. This is noted as a limitation of this study. Given the heterogeneity in chemotherapy regimens in this study, our results are not comparable with prior controlled studies.

The quality of the pancreatic resection and the local radicality are important factors impacting the long-term survival in patients with nonmetastatic PC, especially after a pancreatoduodenectomy.^9,39,40^ With a median OS of 36 months and a PFS of 17 months for R0 resections versus 31 months and 8 months, respectively, for R1 resections, our results mirror previous findings indicating an improved survival associated with R0 resections.^9,19,39,40^ In line with previous evidence, a statistically significant correlation between a higher tumour T classification and improved OS and PFS was detected.^41^

To our knowledge, this series with 162 consecutive surgical NAT patients represents the largest series to date to examine the relationship between cholangitis and postoperative survival in PC patients. Our study took place in a high-volume pancreatic surgery, endoscopy and oncology centre with multidisciplinary team meetings to evaluate tumour resectability, the requirement for NAT and further treatment for our PC patients. The pancreatic surgeons performing the pancreatoduodenectomies and endoscopists performing ERCP were highly experienced, which is supported by the adequate rate of R0 resections and low rate of severe adverse events following surgery and endoscopy.

We note several limitations to our study. Due to the retrospective nature of the study and extensive data collection period, NAT and adjuvant chemotherapy regimens varied. Because patients received NAT and postoperative oncological care in various hospitals, the data on chemotherapy regimens are incomplete. Many patients were followed-up in other hospitals close to their home and, unfortunately, precise data on the time of recurrence were in part unavailable. Furthermore, the number of SEMSes in this study was low, impairing the quality of our comparison of biliary stent types. Unlike the study by Darnell et al.^22^ our analysis was conducted comparing only surgical NAT patients, while patients who initiated NAT but failed to proceed to surgery were excluded. Therefore, our results regarding NAT interruptions are not completely comparable.

## Conclusions

Cholangitis and biliary stent-related interruptions of NAT reduce OS and PFS in patients with pancreatic cancer. To improve the prognosis for PC patients, we should reduce the incidence of cholangitis and minimise the number of NAT interruptions.

### Supplementary Information

Below is the link to the electronic supplementary material.

Supplementary file1 (PDF 68 kb)

Supplementary file2 (DOCX 7 kb)
