# Supplementary material for: Cholangitis and Interruptions of Neoadjuvant Chemotherapy Associate with Reduced Overall and Progression-Free Survival in Pancreatic Cancer
Source: Ann Surg Oncol. 2023 Dec 28;31(4):2621–31. doi: 10.1245/s10434-023-14793-6 (PMC10908635; doi:10.1245/s10434-023-14793-6)
Supplement: Supplementary file 1 — Supplementary file1 (PDF 68 kb) [file 10434_2023_14793_MOESM1_ESM.pdf]

Supplemental Table 1. Neoadjuvant (a) and adjuvant chemotherapy (b) and radiotherapy regimens patients received. Some patients received chemotherapy in other hospital districts and, thus, information regarding the type of chemotherapy received is unknown.

Abbreviations: NAT, neoadjuvant chemotherapy; n, number of patients; GEM, gemcitabine; CAP, capecitabine; CIS, cisplatin; Nab-PTX, nanoparticle albumin-bound paclitaxel; PTX, paclitaxel; ERL, erlotinib; OXA, oxaliplatin; CAR, carboplatin; n/a, not applicable.

a

NAT category	Chemotherapy regimen	n	Cycles	Radiotherapy (n)
GEM alone	GEM	13	3	2
	GEM	2	4	1
	GEM	1	5	0
	GEM	1	8	0
	GEM	1	9	0
GEM combination	GEM + CAP	1	2	0
	GEM + CAP	3	3	0
	GEM + CAP	1	4	0
	GEM + CAP	1	5	1
	GEM + CIS	1	1	0
	GEM + CIS	16	3	1
	GEM + CIS	10	4	3
	GEM + CIS	1	5	0
	GEM + CIS	1	6	0
	GEM + CIS	1	7	0
	GEM + Nab-PTX	1	1	0
	GEM + Nab-PTX	8	2	1
	GEM + Nab-PTX	18	3	4
	GEM + Nab-PTX	3	4	0
	GEM + Nab-PTX	2	5	0
	GEM + Nab-PTX	1	7	1
	GEM + Nab-PTX	1	10	0
	GEM + PTX	3	2	1

	GEM + PTX	2	3	0
	GEM + PTX	2	4	0
	GEM + OXA	1	6	1
	GEM + CIS; GEM	1	3; 3	1
	GEM + Nab-PTX; GEM	1	2; 1	1
	GEM + Nab-PTX; GEM	1	5; 1	0
	GEM; GEM + Nab-PTX	1	1; 2	0
	GEM; GEM + Nab-PTX	1	1; 4	1
	GEM; GEM + Nab-PTX	1	2; 1	0
	GEM; GEM + CIS	1	2; 3	1
FOLFIRINOX	FOLFIRINOX	1	1	0
	FOLFIRINOX	1	2	0
	FOLFIRINOX	8	3	0
	FOLFIRINOX	10	4	0
	FOLFIRINOX	6	5	1
	FOLFIRINOX	2	6	0
	FOLFIRINOX	2	7	0
	FOLFIRINOX	2	8	0
	FOLFIRINOX	2	9	0
	FOLFIRINOX	1	10	1
	FOLFIRINOX lite	1	2	0
	FOLFIRINOX lite	1	3	0
	FOLFIRINOX lite	1	4	0
	FOLFIRINOX lite	2	5	2
	FOLFIRINOX; GEM	1	4; 3	0
	FOLFIRINOX; GEM + Nab-PTX	1	1; 3	0
	FOLFIRINOX; Nab-PTX	1	1; 4	0
Other	CAR + PTX	2	3	1
	Nab-PTX; GEM	1	1; 2	0
Unknown	Unknown	13	n/a	n/a

b

Adjuvant category	Chemotherapy regimen	n
GEM alone	GEM	40
GEM combination	GEM + CAP	23
	GEM + CIS	14
	GEM+ ERL	2
	GEM + Nab-PXT	10
	GEM + PXT	3
FOLFIRINOX	FOLFIRINOX	7
	FOLFIRINOX lite	7
	FOLFIRINOX; GEM	1
Other	OXA + CAP; ERL	1
No adjuvant treatment	n/a	34
Unknown	n/a	20
