# Supplementary material for: Cholangitis and Interruptions of Neoadjuvant Chemotherapy Associate with Reduced Overall and Progression-Free Survival in Pancreatic Cancer
Source: Ann Surg Oncol. 2023 Dec 28;31(4):2621–31. doi: 10.1245/s10434-023-14793-6 (PMC10908635; doi:10.1245/s10434-023-14793-6)
Supplement: Supplementary file 2 — Supplementary file2 (DOCX 7 kb) [file 10434_2023_14793_MOESM2_ESM.docx]

**Supplemental table 2.** Complications related to pancreatoduodenectomy and ERCP.

Abbreviations: DGE: delayed gastric emptying; ERCP: endoscopic retrograde cholangiopancreatography

| **Surgery-related complications needing repeat laparotomy** | **n** |
| --- | --- |
| Pancreaticojejunal anastomosis leakage leading to total pancreatectomy | 2 |
| Hepaticojejunal anastomosis leakage | 2 |
| Infected ascites discoloured by bile (laparotomy due to suspicion of biliary leakage) | 2 |
| Internal hernia | 1 |
| Venous repeat reconstruction due to imminent liver necrosis | 1 |
| Spleen necrosis following venous reconstruction | 1 |
| Surgical drain slipping inside of the abdominal cavity causing peritonitis | 1 |
| **Other complications after surgery** | **n** |
| Major bleeding from arteria hepatica propria needing endovascular treatment | 1 |
| Chyle leak managed conservatively through non-fatty diet | 9 |
| DGE, managed conservatively with gastric tube | 4 |
| DGE, required dilation of the gastrojejunal anastomosis via gastroscopy | 1 |
| Pneumonia | 5 |
| Myocardial infarction | 1 |
| **ERCP-related complications** | **n** |
| Mild post-ERCP pancreatitis needing hospitalization for > 48 hours, managed conservatively | 7 |
| Suspicion of post-ERCP bleeding, no active bleeding was detected upon duodenoscopy, managed conservatively | 2 |
| Cholecystitis during NAT following biliary stent placement, all operated laparoscopically | 4 |
